# The Effect of Payer Status on Survival of Patients With Prostate Cancer

**DOI:** 10.7759/cureus.13329

**Published:** 2021-02-13

**Authors:** Winston Suh, Samip Master, Lihong Liu, Glenn Mills, Runhua Shi

**Affiliations:** 1 Hematology-Oncology, Feist-Weiller Cancer Center, Louisiana State University Health Shreveport, Shreveport, USA

**Keywords:** payer status, prostate cancer, retrospective study, healthcare insurance

## Abstract

Background

Disparities in access to care and proper treatment can have significant implications in patient survival outcome and mortality. This retrospective study of prostate cancer patients from the National Cancer Database (NCDB) between the years 2004 and 2014 and follow-up to the end of 2015 analyzed such effects that variation in payer status might have on outcome.

Methods

This study used the data of 696,321 diagnosed prostate cancer patients from the NCDB for the years 2004 to 2014 and follow-up to the end of 2015 to analyze the effect that payer status would have on prostate cancer survival. Multivariable cox regression was used to study the hazard ratios (HRs) of payer status and other variables along with the Charlson Comorbidity Index to analyze their associated increased risk of death. Statistical software SAS 9.4 for Windows was used to analyze the overall survival (OS) of patients on different insurance plans along with variations in prostate-specific antigen (PSA) levels and treatment type.

Results

When looking at OS, those with private insurance had the greatest overall survivability while those on Medicare were the only ones who reached the median OS. In contrast to those who had private insurance, those who had Medicare, the uninsured, and those with Medicaid demonstrated significantly greater risks of death at 43%, 58%, and 69% increased risk of death, respectively. In addition to payer status, other variables were also significant predictors of OS, including demographic factors (age, race), comorbidities, socioeconomic status (income, education), distance traveled to facility, type of facility, treatment delay, treatment modality, PSA levels at diagnosis, and cancer stage at diagnosis.

Conclusion

Payer status is intricately linked to a number of other variables that might affect survival. Even after adjustment for a number of these factors, insurance status was shown to have a significant effect on prostate cancer survivorship. In contrast to those who had private insurance, those who had Medicare, the uninsured, and those with Medicaid demonstrated significantly greater risks of death at 43%, 58%, and 69% increased risk of death, respectively. Studies have suggested that those without insurance or on Medicaid are less likely to undergo screening and have worse health-related quality of life, while those on Medicare may be deterred from continuing treatment due to high out-of-pocket costs. However, the complete mechanism behind the improved survivorship of those on private insurance is unclear. The effect of payer status on quality of life may be an interest that needs to be further studied. Further research will be required to provide definite reasons for these observations and mediation analysis of other factors could prove to be valuable.

## Introduction

Prostate cancer is the second leading cause of cancer death in American men after lung cancer and the most common cause of cancer in American men after skin cancer [1]. With data from 2012-2016, while the most common newly diagnosed cases of prostate cancer are those in the age group of 65-74 years, the greatest ratio of deaths (33.4%) is within the age group of 75-84 years [2]. Studies show this may be related to treatment variations with older men more likely to receive hormone therapy rather than a curative local therapy [3]. Incidence rates for blacks, whites, and Asian/Pacific Islanders were 176.7, 101.9, and 55.6 new cases per 100,000 men per year, respectively. Mortality rates per 100,000 men per year are in a similar order, with blacks (38.9), whites (18.0), and Asian/Pacific Islanders (8.6) [2]. Such racial differences in statistics can be explained by numerous factors including later stages at diagnosis, lower socioeconomic status, and differences in treatment [4,5]. As with any malignancy, prostate cancer has the best survival rates when diagnosed and treated in the early stages [2], and, likewise, a worse prognosis has been shown with later stages of diagnosis [4]. The higher stage and grade of diagnosis that have been cited as key explanatory factors in cancer survival are intrinsically linked to socioeconomic factors, which include lower income and less education [6,7].

Other notable factors that affect prostate cancer survival include the presence of comorbidities [5,6], treatment type [8], and treatment delays [9]. Facility type has been also shown to influence outcome; specifically, worse outcomes have been shown to be associated with community care centers [10]. Studies have shown that traveling to secondary facilities, perhaps for secondary opinions, may have little effect in treatment choices but may also add value and new interpretations of tests for proper patient care [11]. Longer distances traveled to a primary facility have been linked with lower mortality rates [12], while access to proper, definitive treatments has also been shown to be key in initial treatment of cancers [13]. And while prostate-specific antigen (PSA) has long been used as a marker of biochemical activity and has shown to have some level of clinical validity and predictive risk of prostate cancer-specific death [14,15], other studies show this relationship might not be so clear, especially with higher PSA levels [16]. Most relevant to this study is the growing body of evidence that shows payer status, or insurance, to be important in the management and outcomes of cancer patients [10,17-20].

Most insurance plans today can be classified as private or public (Medicaid and Medicare), with patients in each group meeting certain characteristics. While Medicaid provides coverage to low-income families, qualified children, pregnant women, parents, seniors, and individuals with disabilities or those who are medically needy, Medicare provides coverage for people who are 65 years or older, certain younger people with disabilities, or people with end-stage renal disease. These are a few of the many features that can affect a patient’s prognosis. So far, there have been limited studies to assess payer status effect on prostate cancer survivorship. Current research assessed the effect of insurance on survival of patients with prostate cancer.

## Materials and methods

This study used the data of 696,321 diagnosed prostate cancer patients from the de-identified the National Cancer Database (NCDB) file for the years 2004-2014 and follow-up to the end of 2015 to assess the effect that payer status would have on prostate cancer survival. The primary predictor variable in this study was insurance. Additional variables addressed included age, race, the presence of comorbidities, income, education, distance traveled to primary facility, facility type, diagnosing/treating facility, treatment delay from diagnosis, PSA levels, types of treatment, and the diagnosed tumor stage.

Payer status, also known as insurance status, was subdivided into those who were uninsured and those who had private insurance, Medicaid, or Medicare. Age at diagnosis was grouped based on ranges of 18-54, 55-64, 65-74, or 75+ years. Race included identifications of white, black, or Asian. The Charlson Comorbidity Index indicated whether any comorbidity was present with the cancer (“yes”) or not (“no”). Zipcode level, income, and education were used in this research. Annual household income was subdivided into <US$30,000, US$30,000-35,000, US$36,000-45,000, and > US$46,000. Education ranges of <14%, 14-19.9%, 20-28.9%, and ≥29% indicated those who did not have high school education. The distance traveled to the facility was identified as being less or more than 30 miles.

Facility type was divided based on the NCDB’s categorization of integrated network cancer programs, academic/research programs, community cancer programs, or comprehensive community cancer programs. Diagnosing/treating facility indicates whether the patient was diagnosed and treated at the same facility they initially presented at. “Treatment Started from Diagnosis” indicates the delay in treatment from diagnosis and includes day ranges of 0-11, 12-23, 24-39, and 40+ days. PSA levels, measured in ng/mL units, were categorized as being within the ranges of 0.1-3.9, 4-9.9, 10-19.9, or 20-99. Tumor staging was categorized as I, II, III, and IV based on the American Joint Committee on Cancer staging system. Treatment variations were grouped into three categories: either hormone therapy alone, radiation and hormone therapy, or a combination of radiation, hormone therapy, and surgery.

Chi-square test was used to test the association between payer status and stage at diagnosis. The log-rank test was used to assess various factors on the survival of prostate cancer. Multivariate cox regression was used to study the effect of payer status on survival of prostate cancer while controlling for other variables. Statistical software SAS 9.4 for Windows (SAS Institute Inc., Cary, NC, USA) was used for all data. All p-values <0.05 or confidence intervals (CIs) of hazard ratio (HR) not including 1 were considered statistically significant in this research.

## Results

This study analyzed 696,321 prostate cancer patients from the NCDB (Table 1). Among the 696,321 patients, a majority of patients had private insurance (50.30%) followed by those with Medicare (45.70%), Medicaid (2.30%), and no insurance (1.69%). Most patients (74.30%) were within the age range of 55-74 years at diagnosis. Patients with household income of more than US$46,000 per year comprised 43.70% of the total, and 76.24% of total patients traveled less than 30 miles to reach their initial facility for work-up. PSA levels at presentation were distributed with ranges of 0.1-3.9, 4-9.9, 10-19.9, and 20-99 ng/mL for 15.99%, 59.04%, 12.96%, and 12.00% of patients, respectively. Among these patients, tumor AJCC (American Joint Committee on Cancer) staging was observed to have a distribution of 8.13%, 74.18%, 11.21%, and 6.47% for stage I, II, III, and IV cancers, respectively. The majority of patients (56.06%) received treatment of a combination of radiation, hormone therapy, and surgery.

**Table 1:** Characteristics of prostate cancer patients from the NCDB between 2004 and 2014 PSA, prostate-specific antigen; AJCC, American Joint Committee on Cancer; NCDB, National Cancer Database

Factor	Level	n	%
Age, years	18-54	90233	12.96
	55-64	261246	37.52
	65-74	256076	36.78
	75+	88766	12.75
Race	White	582886	83.71
	Black	99634	14.31
	Asian	13801	1.98
Comorbidity index	No	587022	84.30
	Yes	109299	15.70
Insurance	Private	350279	50.30
	Medicaid	16031	2.30
	Medicare	318251	45.70
	Uninsured	11760	1.69
Income	US$30,000	83913	12.05
	US$30,000-35,000	117737	16.91
	US$36,000-45,000	190365	27.34
	>US$46,000	304306	43.70
Education (without high school)	<14%	279967	40.21
	14-19.9%	164362	23.60
	20-28.9%	151460	21.75
	≥29%	100532	14.44
Distance traveled to facility	<30 miles	530848	76.24
	30+ miles	165473	23.76
Facility type	Integrated Network Cancer Program	72132	10.36
	Academic/Research Program	257780	37.02
	Community Cancer Program	55604	7.99
	Comprehensive Community Cancer Program	310805	44.64
Diagnosing/treating facility	Same facility	353379	50.75
	Different facility	342942	49.25
Treatment started from diagnosis (days)	0-11	65254	9.37
	12-23	47884	6.88
	24-39	82472	11.84
	40+	500711	71.91
PSA levels (ng/mL)	0.1-3.9	111359	15.99
	4-9.9	411113	59.04
	10-19.9	90267	12.96
	20-99	83582	12.00
Treatment	Hormone therapy	31692	4.55
	Radiation + hormone therapy	274277	39.39
	Radiation + hormone therapy + surgery	390352	56.06
AJCC stage	I	56639	8.13
	II	516548	74.18
	III	78082	11.21
	IV	45052	6.47

There was a strong association between prostate cancer stage and payer status (p < 0.0001). With regard to both cancer stage and payer status (Table 2), regardless of the insurance type, the majority of patients presented and were diagnosed with stage II prostate cancer. The uninsured and Medicaid patients presented with more than 28% of stage III and stage IV cancers. Finally, those with private insurance and Medicare were diagnosed only with about 17% stage III and stage IV cancers.

**Table 2:** Relationship between insurance and stage at diagnosis of prostate cancer

	Stage	I		II		III		IV	
		n	%	n	%	n	%	n	%
Insurance	Uninsured	844	6.64	7843	61.72	1532	12.06	2488	19.58
	Private	30807	8.15	282935	74.84	47078	12.45	17243	4.56
	Medicaid	1352	7.75	11153	63.91	1962	11.24	2985	17.10
	Medicare	27460	8.00	256285	74.62	33639	9.79	26078	7.59

**Figure 1:**
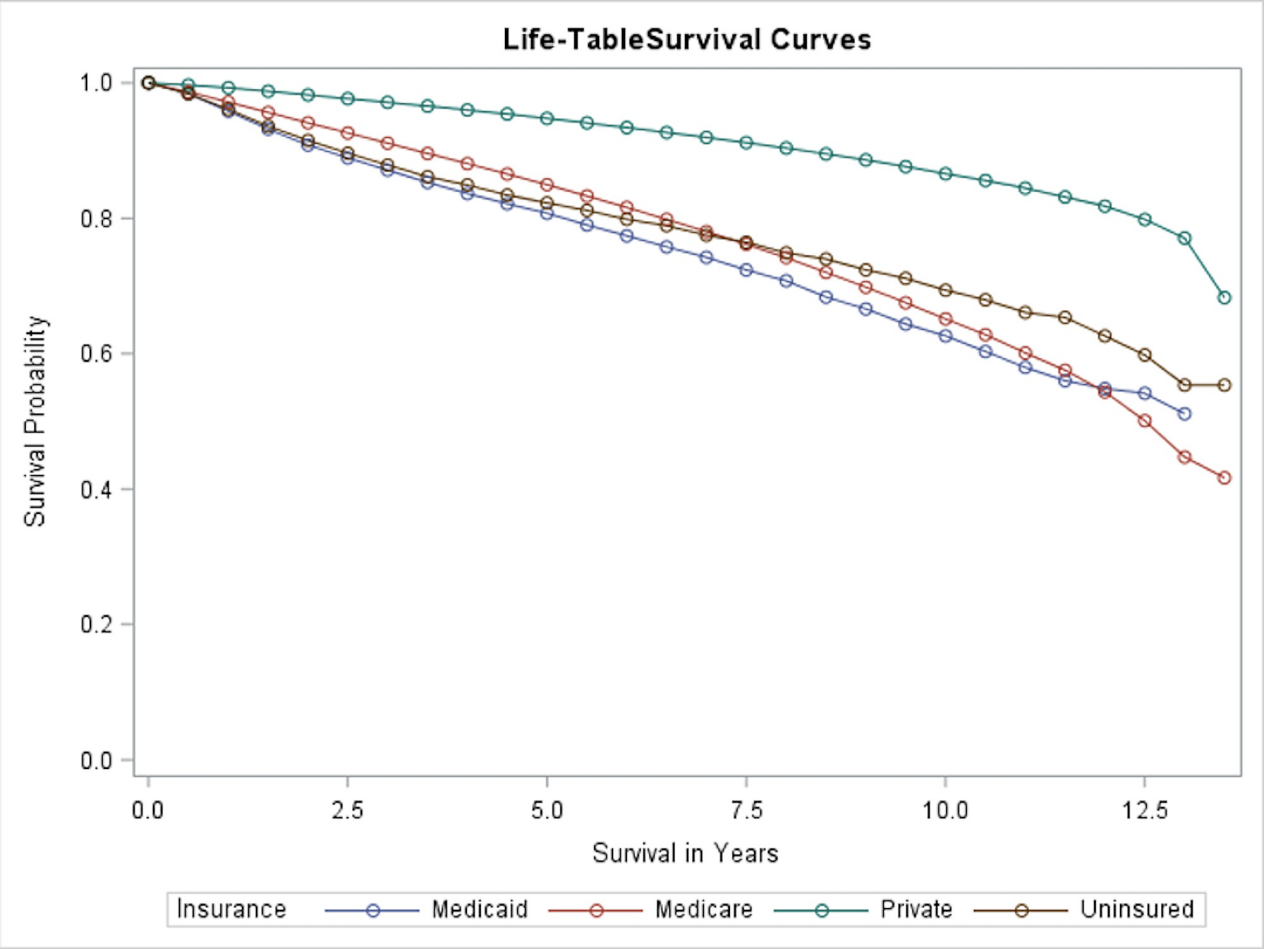
Overall survival of patients with different insurance

Payer status was a significant predictor of survivorship in prostate cancer patients. In univariate analysis, patient survival according to their payer status was assessed as shown in Figure 1. It was shown that the patients with private insurance had a much better survival as compared to those with other insurance status. The median overall survival (OS) for Medicare patients was 12.5 years. For other groups, the median OS was not reached and was more than 13.5 years. In summary, those who had the best OS probabilities were those on private insurance, while those who had the worst were on Medicare.

There was a strong association between prostate cancer stage and PSA level (p < 0.0001). With regard to both cancer stage and payer status (Table 3), regardless of the PSA level, the majority of patients presented and were diagnosed with stage II prostate cancer. The higher the PSA level, the more likely patients were to present with stage III and stage IV cancers.

**Table 3:** Relationship between PSA level and stage at diagnosis of prostate cancer PSA, prostate-specific antigen

	Stage	I		II		III		IV	
		n	%	n	%	n	%	n	%
PSA (ng/mL)	0.1-3.9	13057	10.75	94828	78.1	10398	8.56	3135	2.58
	04-9.9	43128	9.61	349633	77.88	47906	10.67	8264	1.84
	10-19.9	2756	2.76	73968	74.07	15515	15.54	7624	7.63
	20-99	2385	2.59	47872	51.89	11410	12.37	30593	33.16

**Figure 2:**
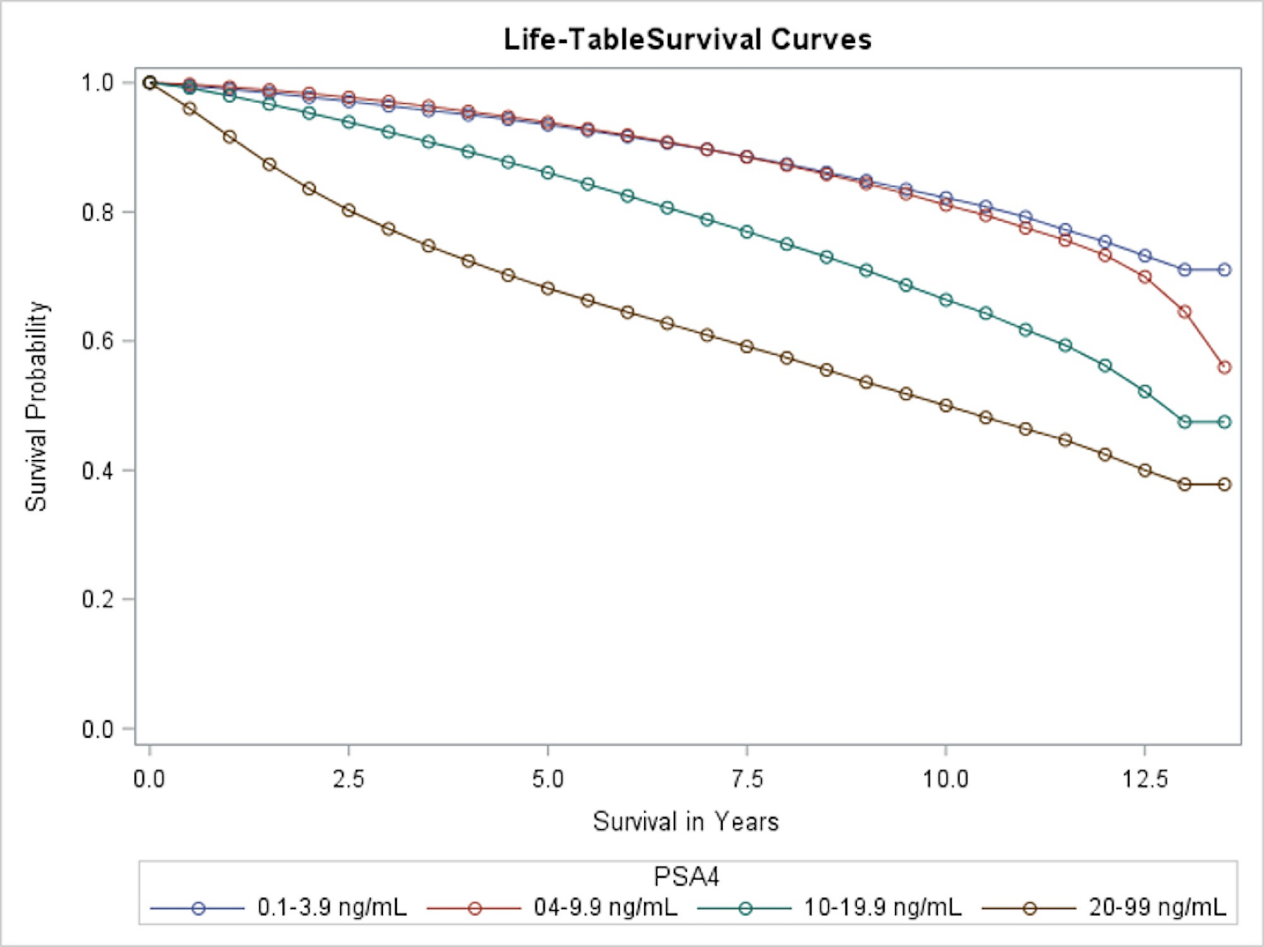
Overall survival of prostate cancer patients with different PSA levels PSA, prostate-specific antigen

PSA levels at diagnosis were also shown as a significant predictor of prostate cancer survival. In univariate analysis, patient survival according to their PSA levels at diagnosis was assessed, as shown in Figure 2. A direct relationship was shown with the best survival at the lowest PSA range and increasingly worse survival with higher PSA ranges. The median OS of patients in the 10-19.9 and 20-99 ng/mL ranges was 13 and 10 years, respectively. For other groups, the median OS was not reached and was more than 13.5 years. In summary, those who had the best OS probabilities were those with initial PSA ranges of 0.1-3.9 ng/mL, while those who had the worst had initial PSA ranges of 20-99 ng/mL.

**Figure 3:**
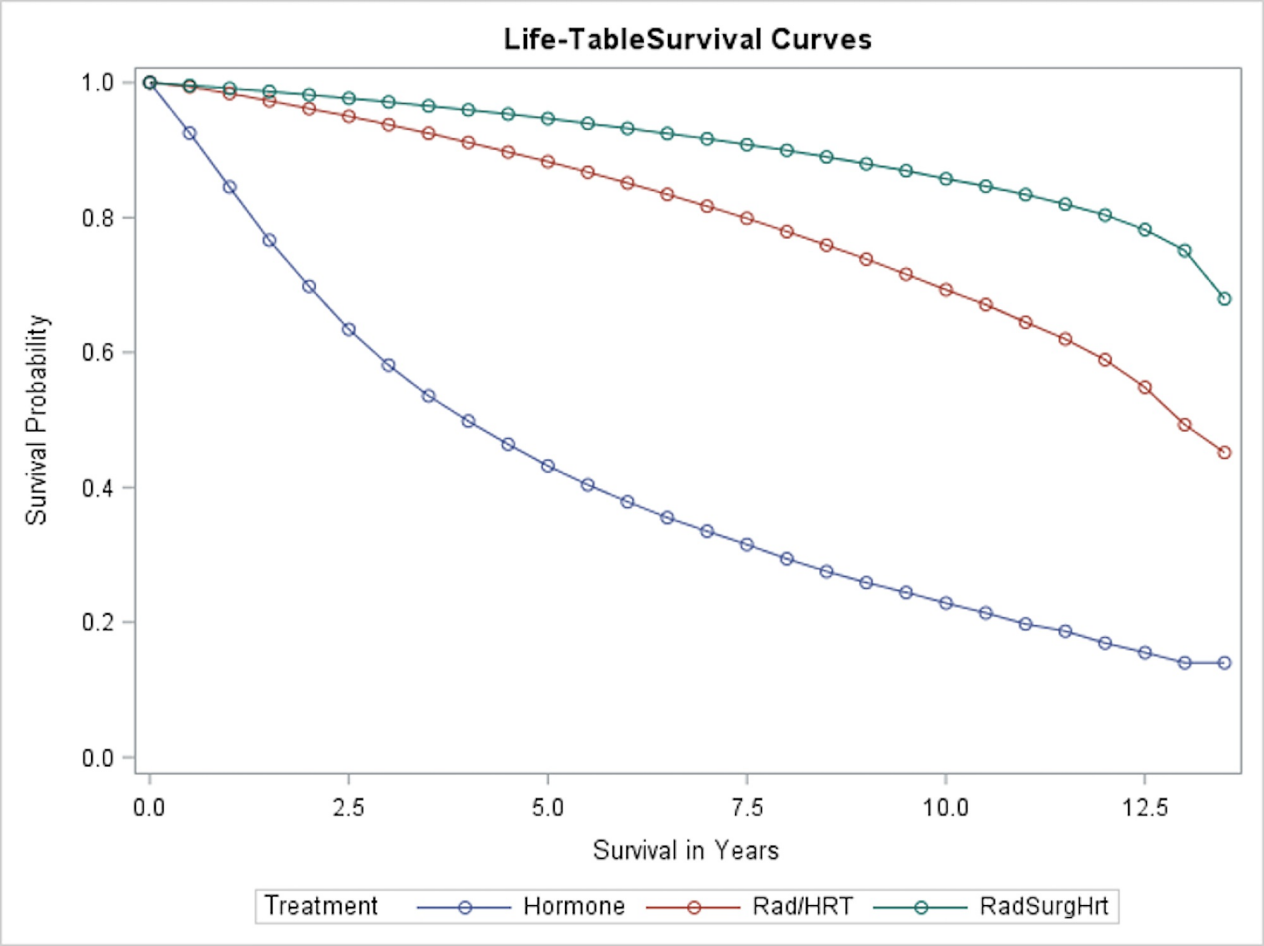
Overall survival of patients with different treatments

Treatment, as expected, was also a significant predictor of OS. Univariate analysis revealed that patient survival was associated with different treatment plans, as shown in Figure 3. The median OS of patients who received only hormone therapy was four years, while the patients who received a combination of hormone therapy and radiation had a median OS of 13 years. The median OS was not reached and was more than 13.5 years for the group that received hormone therapy, radiation, and surgery. Without adjusting for other factors, such as stage, the addition of radiotherapy improved survivorship, and the further addition of surgery proved to have the best OS.

Adjustment was not performed during the univariate analysis of the effect of payer status and PSA levels, as well as treatment on the survival of prostate cancer patients. The effect might not be true due to the effect of covariates on survival. Therefore, a multivariate Cox proportional hazards (CPH) model was used to evaluate the effect of payer status on prostate survival (Table 4) adjusting for other covariates. In contrast to those who had private insurance, those who had Medicare, the uninsured, and those with Medicaid demonstrated significantly increased risks of death at 43%, 58%, and 69%, respectively.

**Table 4:** Multivariable Cox regression of overall survival of predictor effect PSA, prostate-specific antigen; AJCC, American Joint Committee on Cancer; NCDB, National Cancer Database

Factor	Level	HR	Lower	Upper
Age, years	18-54	1.00		
	55-64	1.28	1.24	1.32
	65-74	1.69	1.64	1.75
	75+	3.54	3.42	3.66
Race	White	1.00		
	Asian	0.78	0.74	0.82
	Black	1.07	1.05	1.09
Comorbidity index	No	1.00		
	Yes	1.59	1.57	1.62
Insurance	Private	1.00		
	Medicaid	1.69	1.63	1.75
	Medicare	1.43	1.40	1.46
	Uninsured	1.58	1.51	1.66
Income	> US$46,000	1.00		
	< US$30,000	1.19	1.16	1.22
	US$30,000-35,000	1.13	1.11	1.16
	US$36,000-45,000	1.09	1.07	1.11
Education (without high school)	<14 %	1.00		
	14-19.9%	1.11	1.09	1.13
	20-28.9%	1.15	1.13	1.18
	≥29%	1.18	1.15	1.21
Distance traveled to facility	<30 miles	1.00		
	30+ miles	0.90	0.88	0.91
Facility type	Integrated Network Cancer Program	1.00		
	Academic/Research Program	0.92	0.90	0.94
	Community Cancer Program	1.14	1.11	1.17
	Comprehensive Community Cancer Program	1.04	1.01	1.06
Diagnosing/treating facility	Same Facility	1.00		
	Different Facility	0.97	0.96	0.98
Treatment started from diagnosis (days)	0-11 days	1.00		
	12-23	0.69	0.68	0.71
	24-39	0.60	0.59	0.61
	40+	0.51	0.51	0.52
PSA levels (ng/mL)	0.1-3.9	1.00		
	4-9.9	0.95	0.93	0.97
	10-19.9	1.30	1.27	1.33
	20-99	1.53	1.49	1.56
Treatment	Hormone therapy	1.00		
	Hormone therapy + radiation	0.70	0.69	0.72
	Hormone therapy + radiation + surgery	0.42	0.41	0.43
AJCC stage	I	1.00		
	II	1.11	1.07	1.15
	III	1.54	1.48	1.61
	IV	5.35	5.13	5.57

In addition to payer status, multivariate CPH analysis demonstrated that other variables were significant predictors of OS. In specific, analysis of PSA levels, when compared to the control range of 0.1-3.9 ng/mL, showed increased HRs and, thus, increased risk of death in ranges of 10-19.9 ng/mL (1.30) and 20-99 ng/mL (1.53) but a lower risk with those who had PSA levels in the 4-9.9 ng/mL range (0.95; 95% CI: 0.93-0.97). The reason for this was unclear. When looking at treatment with hormone therapy as the control, those who also received radiation in addition to hormone therapy were 30% less likely to die, while those who received a combination of radiation, surgery, and hormone therapy were 58% less likely to die.

Adjusting for other factors, age, race, comorbidities, and tumor stage also showed significant predictors for prostate cancer survival. In relation to the age range of 18-54 years, those who were 75 years and older had a 3.54 times greater chance of dying from prostate cancer. With relation to race, Asians had a 22% lower chance of dying in comparison to whites. Those who presented with any additional comorbidities had a 59% greater chance of death. As compared to stage I, HR increased to 1.11, 1.54, and 5.35 for stages II, III, and IV metastatic cancers, respectively.

Other significant predictors include income, education, facility type, travel distance, and treatment delay. Having less than a US$30,000 yearly household income increased chance of death by 19% vs. those who had a > US$46,000 income. Those in the 20-28.9% and ≥29% groups who did not attend high school had 15% and 18% increased chances of deaths, respectively, as opposed to those in the <14% group who did not attend high school. Those who were treated at a community cancer program had a 14% greater risk of death than those treated at an integrated network cancer program, while those who traveled more than 30 miles to their facility had a 10% decreased risk of death. Finally, a greater delay before starting treatment led to a lower risk of death, all the way to a 49% chance of decreased death with a 40+ day treatment delay.

## Discussion

In our research, we demonstrated payer status to be strongly associated with prostate cancer OS. In Figure 1, the higher OS of those on private insurance versus those with different insurance status can be seen. Even with factors controlled for (Table 4), the greater risk of death in those who were uninsured, on Medicare, or on Medicaid compared to those with private insurance is made clear with the higher HRs. This finding between payer status and survivorship has similarly been seen with other types of cancer [10,20,21]. There could be a number of explanations for this finding.

In Ayanian et al.’s study of unmet health needs of uninsured adults in the United States, it was found that those who were uninsured, whether long-term or short-term, were not only less likely to see a physician than the insured due to the cost, but they were also less likely to have routine checkups and cancer screenings overall [22]. Similar findings have been found in studies of other cancers where low rates of screening have been reported by subgroups such as those with no health care coverage or those on Medicaid [21,23]. With reduced access to cancer screening or physician visits, patients lacking health insurance or on Medicaid have been shown to have greater odds of a late or less favorable stage of prostate cancer diagnosis [17-19], as seen in Table 2 of our study.

Another interesting theory is put forth by Smith et al. who studied a cohort of 523 adolescents and young adults and observed that respondents reported poor health-related quality of life in the form of poor physical (e.g., fatigue) and emotional functioning after cancer diagnosis. This was especially true for those with, among other variables, a lack of health insurance [24]. This suggests possible cost or treatment barriers and difficulty with adherence to post-treatment screenings such as biopsies, scans, and PSA tests, thus leading to worse outcomes. A review of the literature has also revealed that the HRs seen with the Medicare population could be connected to a greater tendency toward hospitalization and resulting high out-of-pocket costs [25], ultimately deterring patients from continuing their treatment. More specifically, our study demonstrates a clear discrepancy in survivorship between those on Medicaid who had a 69% increased risk of death and those on Medicare who only had a 43% increased risk of death. This finding seen in many other studies might be related to variables such as stage at diagnosis [26] and access to care [27]. However, distance traveled, stage at diagnosis, and other socioeconomic factors were adjusted in the current research while assessing the effect of payer status on prostate cancer survival.

Table 3 demonstrates that, in addition to payer status, other variables such as demographic factors (age, race) are significant to survival. In particular, older age was shown to be associated with higher risk of death [3] and being of Asian race was associated with lower risk of death [28]. Consistent with the current literature, a higher socioeconomic status (in this case, education and income) has been linked to a lower mortality after prostate cancer diagnosis [6,7], while treatment at community cancer centers has been shown to be associated with a worse outcome [10]. In addition, comorbidities have repeatedly been shown to have adverse effects on prostate cancer outcomes [6,29], while the drastically decreased HRs when using a combination of radiation and/or surgery with hormone therapy indicate the importance of using polytherapy as opposed to monotherapy [8]. This becomes especially relevant when dealing with castrate-resistant cancer in which cancer progresses even with hormone deprivation.

PSA levels at diagnosis also lead to an increased risk of death and were linked to a decreased OS. PSA-based screening has been shown to be an appropriate method for prostate cancer screening [15]; therefore, this relationship might indicate a poor survival associated with increased tumor burden. Furthermore, there is a clear, direct, and expected relationship between a higher tumor stage and HRs [4]. An interesting relationship is shown with treatment delay where an increasing delay resulted in decreased HRs. This may be related to the fact that studies have shown active surveillance to yield better prostate-cancer specific mortality when compared with immediate radical treatments for lower-risk prostate cancers [9,30].

The effect of payer status on survivorship has been seen in related cancer studies [10,20,21] and was shown to be significant in our study. We were able to illustrate certain reasons for these discrepancies with current literature, but the mechanism of payer status alone on survivorship remains unclear and may benefit with future mediation analyses of other hypothesized variables.

Despite certain strengths to this study, such as a large sample size and use of a multivariate analysis, there exist certain limitations. First, this was a retrospective study; therefore, certain selection bias may have occurred in gathering patient data. And while this research assessed overall prostate cancer survival, cause-specific survival could not be assessed due to limitations of data from the NCDB. Second, zip code, education, and income variables were collected as opposed to an individual education and income. And finally, payer status was only assessed at diagnosis. Changes to insurance post-diagnosis or during treatment were not documented.

## Conclusions

Payer status is intricately linked to a number of other variables that might affect survival. Even after adjustment for a number of these factors, insurance status was shown to have a significant effect on prostate cancer survivorship. In contrast to those who had private insurance, those who had Medicare, the uninsured, and those with Medicaid demonstrated significantly greater risks of death at 43%, 58%, and 69% increased risk of death, respectively. Studies have suggested that those without insurance or on Medicaid are less likely to undergo screening and have worse health-related quality of life, while those on Medicare may be deterred from continuing treatment due to high out-of-pocket costs. However, the complete mechanism behind the improved survivorship of those on private insurance is unclear. The effect of payer status on quality of life may be an interest that needs to be further studied. Further research will be required to provide definite reasons for these observations. and mediation analysis of other factors could prove to be valuable.
